# A New Measure of Reading Habit: Going Beyond Behavioral Frequency

**DOI:** 10.3389/fpsyg.2016.01364

**Published:** 2016-09-08

**Authors:** Fabian T. C. Schmidt, Jan Retelsdorf

**Affiliations:** Leibniz Institute for Science and Mathematics EducationKiel, Germany

**Keywords:** reading habit, self-report habit index for reading (SRHI-R), reading frequency, habit measure, reading achievement

## Abstract

Reading habit is considered an important construct in reading research as it serves as a significant predictor of reading achievement. However, there is still no consensus on how to best measure reading habit. In recent research, it has mostly been measured as behavioral frequency; this approach neglects the fact that repeated behavior does not cover the broad content of habitual behavior—such as automaticity and the expression of one’s identity. In this study, we aimed to adapt a 10-item scale on the basis of the Self-Report Habit Index by Verplanken and Orbell (2003) that is comprehensive but still economical for measuring reading habit. It was tested by drawing on a sample of *N* = 1,418 upper secondary school students. The scale showed good psychometric properties and the internal and external validity was supported. Moreover, the scale predicted reading achievement and decoding speed over and above reading frequency. The implications of an elaborated but still economical way of measuring reading habit are discussed giving new impetus on research on reading habit, challenging conventional approaches of traditional measures.

## Introduction

Reading habit is considered as an important variable in reading research. However, researchers often use the term habit synonymously to behavioral frequency. Thereby, the concept of habit often forms the theoretical foundation, even if in a particular study labels such as reading activity, behavior or frequency are used. Thus, it might be promising to adapt the elaborated habit concept by Verplanken and Orbell (2003) to reading research. We aimed to introduce a broader unidimensional conception of habit to reading research involving behavioral frequency, automaticity and the expression of one’s identity. We adapted the Self-Report Habit Index (SRHI, Verplanken and Orbell, 2003) introducing the Self-Report Habit Index for Reading (SRHI-R). We aimed to provide an economic measure that is easy to administer and can be used in typical research designs in reading research such as longitudinal large-scale assessments. In this study, we assessed the psychometric properties of the SRHI-R and investigated some aspects of its validity. Finally, we tested for the incremental validity over and above reading frequency. We will first review the conception of habit presented by Verplanken and Orbell (2003) before, we describe its application to reading.

Verplanken and Aarts (1999) define habit as “learned sequences of acts that have become automatic responses to specific cues, and are functional in obtaining certain goals or end states” (p. 104). For a detailed discussion of the habit construct see Bernacer et al. (2015). Following Verplanken and Orbell (2003) habit is best described as a unidimensional construct, e.g., it can be used as a compound measure assessing reading habit, covering several aspects characterizing habitual behavior. First, the history of repetition is important because a certain behavior has to have been executed successfully and repeatedly in the past to form a habit. Second, the expression of one’s identity is relevant because habits not only form the behavioral repertoire but also “reflect a sense of identity or personal style” (p. 1317)—partly because habits are part of how we organize our everyday life. Finally, automaticity is an important component of habit comprising three aspects (Bargh, 1994): First, lack of awareness refers to a lack of a conscious intent or not being aware that a process is instigated. Second, mental efficiency suggests that habits free mental capacities and thus enable us to shift attention to additional processes. Third, lack of control means that habitual behavior is only controllable to a certain degree, which may be easily understood when thinking of how hard it can be to break a bad habit (Verplanken and Faes, 1999; Adriaanse et al., 2014; Güell and Núnez, 2014) or to develop new habits (Page and Page, 2014). It is important to note that habits develop by successful and satisfying repetition in stable contexts, whereby not only repetition but also automatization of the behavior is essential for the definition of habit (Verplanken et al., 2005).

Verplanken and Orbell (2003) developed the SRHI, a short measure of habit strength accounting for these aspects of habit. The SRHI showed excellent internal consistency; content, discriminant, and predictive validity were good for a variety of different behaviors such as health behavior, social chatting, transportation mode choices, and mental habits (Verplanken, 2006).

In reading research habit has mostly been measured as reading frequency, reading amount, or reading activity. Thereby, the particular label used is not always equivalent to the meaning of a construct. In fact, many researchers at least implicitly aim to assess reading habit. For example, the Reading Activity Inventory claims to also assess “reading habit” (p. 10, Guthrie et al., 1994) without defining this term at all. Our aim was to apply the aforementioned conceptualization of habit to reading providing a more elaborated theoretical framework for investigating reading habit.

There are several approaches claiming to measure reading habit. Questionnaires are frequently used but often consist of only one to a few items assessing reading frequency or directly asking about reading habit (e.g., Applegate and Applegate, 2004; OECD, 2010). Measures of behavioral frequency have been criticized as described above. Moreover, Verplanken et al. (2005) argue that once a habitual behavior is established, repetition is needed in order to sustain it as a habit. Asking for enjoyment might mix up reading motivation and habit to some extent. Moreover, it can be difficult to retrieve episodic memories if asked for behavioral frequency in the past (e.g., “How often/long did you read last week?”), or when the habit concept being questioned is not conceptualized clearly (Verplanken, 2010). Finally, one-item measures are “notoriously unreliable” (Verplanken, 2010, p. 72).

Other approaches used diary studies, reading log books, or task analyses to assess reading habit (Taylor et al., 1990; Allen et al., 1992). Diary methods or log books seem to be a promising approach measuring habit. However, these approaches also only consider reading frequency and they are quite time-consuming for participants and researchers, and can often not be implemented in research designs, such as longitudinal large-scale assessments (cf. Wigfield and Guthrie, 1997). Moreover, Verplanken (2006) argues that the diary method makes participants more aware of their behavior and thus—at least for social desirable behavior—may lead to an overestimation of behavioral frequency and in turn to an overestimation of the actual habit. Finally, diary methods as measures of reading frequency in general may lead to a misinterpretation for example when a child is asked to read daily by his or her parents.

There are manifold approaches for measuring whether individuals read often and broadly. However, these instruments are mostly restricted to behavioral frequency. Following Verplanken (2010) such instruments are not valid measures of habit since they “fail to measure any other aspect of habit than behavioral frequency” (p. 72).

## The Present Investigation

The aim of our study was to introduce a broader habit conceptualization in reading research as well as to validate the SRHI-R as an efficient measure of reading habit. We adapted the SRHI (Verplanken and Orbell, 2003) to reading. Following Verplanken et al. (2005), we only adapted ten out of twelve items, since for some behaviors single items of the SRHI may be less useful for measuring the according habit.

First of all, we tested the dimensionality of the SHRI-R. Habit in its essence is understood as a unidimensional psychological construct even though it comprises aspects of the history of repetition, automatization, and identity. Thus, we expected a unidimensional factor structure for the SRHI-R that has been empirically supported for the original SRHI (Verplanken and Orbell, 2003; Verplanken et al., 2005). Second, we tested psychometric properties (reliability, item difficulties, item selectivity) before, we investigated some aspects of validity—measurement invariance, correlations with external criteria, and incremental validity compared to behavioral frequency.

Regarding measurement invariance, we focused on gender and students in different thematic study-profiles as relevant subpopulations. Thematic study-profiles, namely esthetics, language, science, social science, or sports represent the main field of study in upper secondary schools in some federal states of Germany.

We considered five theoretically derived external criteria for which, we expected the following correlations. First, we expected high positive correlations between the SRHI-R and traditional measures of reading frequency. Second, we expected positive correlations between the SRHI-R and intrinsic reading motivation and reading self-concept as important aspects of reading motivation (cf. Retelsdorf et al., 2011). Third, we expected differential correlations between the SRHI-R and the proximal verbal self-concept versus the distal mathematics self-concept as a test of convergent and discriminant validity. Fourth, in a similar vein, we expected differential correlations with school grades in the first language (German) and mathematics. Fifth, we expected positive correlations with reading achievement and decoding speed since reading habit should lead to a higher extent of reading practice and, thus, to an enhancement of reading (Mol and Bus, 2011). Finally, we tested if the SRHI-R explains additional variance in reading achievement and decoding speed over and above measures of behavioral frequency.

## Materials and Methods

### Sample

Our sample stemmed from the LISA-project (German: “Lesen in der Sekundarstufe” [Reading in secondary school]), which mainly deals with individual and contextual determinants of reading achievement (e.g., Retelsdorf et al., 2012, 2014). We drew on data from the fifth wave (previously the SRHI-R was not included) comprising *N* = 1,418 upper secondary school students in 11^th^ Grade at academic-track schools (54% female; age: *M* = 17.24, *SD* = 0.67). More details about the LISA sample are provided elsewhere (Retelsdorf et al., 2012).

### Measures

#### Reading Habit

We adapted the SRHI by Verplanken and Orbell (2003) for reading and translated it into German, resulting in a 10-item measure of reading habit. All items are provided in **Table 1**. Students rated their agreement on a five-point Likert-type scale anchored at 1 (‘totally disagree’) and 5 (‘totally agree’). The scale was introduced by the question “How do you feel about reading in your leisure time?” and the prefix “Reading in my spare time is something…”.

**Table 1:** Means, standard deviations, item selectivities, item difficulties, factor loadings from EFA, and standardized factor loadings from CFA for the unidimensional model of the SRHI-R.

	Reading in my spare time is something…	*M*	*SD*	*r*_it_	*p*_corr_	Fl*_n_*_=419_	λ*_ν_*_=999_
1	I do frequently.	3.36	1.33	0.85	0.52	0.894	0.897
2	I do automatically.	3.15	1.36	0.87	0.47	0.883	0.900
3	I do without having to consciously remember.	3.37	1.35	0.78	0.52	0.801	0.782
4	that makes me feel weird if I do not do it.	2.78	1.37	0.81	0.38	0.856	0.805
5	I do without thinking.	3.16	1.32	0.80	0.47	0.829	0.768
6	that would require effort not to do.	3.03	1.37	0.86	0.44	0.875	0.836
7	that belongs to my daily routine.	2.81	1.37	0.83	0.39	0.838	0.844
8	I do not need to think about doing.	3.08	1.39	0.72	0.46	0.750	0.704
9	that is typically “me.”	2.91	1.37	0.88	0.41	0.887	0.906
10	I have been doing for a long time.	3.57	1.44	0.80	0.59	0.828	0.830

*r_it_, part-whole-corrected item selectivities; *p*_corr_, corrected item difficulties; Fl, factor loadings on the first factor from the pattern matrix of the EFA; λ, standardized factor loadings from CFA. Responses were made on a 5-point Likert scale ranging from (1) strongly disagree to (5) strongly agree.*

#### Reading Frequency

We used a one item frequency measure (‘How often do you read for enjoyment’) that has been used in several large-scale assessments such as the Progress in International Reading Literacy Study (PIRLS; Bos et al., 2007) or the Programme for International Student Assessment (PISA, OECD, 2010). Answers were rated on a five-point Likert-type scale: 1 = ‘I don’t read for enjoyment,’ 2 = ‘up to 30 min daily,’ 3 = ‘30 min to 1 h daily,’ 4 = ‘1–2 h daily,’ 5 = ‘more than 2 h daily.’

#### Intrinsic Reading Motivation

Intrinsic reading motivation (five items, e.g., ‘I enjoy reading books,’ α = 0.94) was measured with the according subscale from the Habitual Reading Motivation Questionnaire (Möller and Bonerad, 2007). For the scale, the answers were rated on a four-point Likert-type scale anchored at 1 (‘does not apply to me’) and 4 (‘applies to me’).

#### Academic Self-Concepts

Reading self-concept was assessed with four items, (e.g., ‘Generally, understanding texts is easy for me,’ α = 0.75) from the Habitual Reading Motivation Questionnaire (Möller and Bonerad, 2007). The answers were rated on a four-point Likert-type scale anchored at 1 (‘does not apply to me’) and 4 (‘applies to me’). Items for assessing verbal (α = 0.87) and mathematics (α = 0.90) self-concept were taken from the short form of the Self-Description Questionnaire by Marsh (1992). For both domains, the three items (e.g., ‘Mathematics/German is one of my best subjects’) were rated on a four-point Likert-type scale with anchors at 1 (‘not true at all’) and 4 (‘completely true’).

#### School Grades

Students’ grades were collected from their latest report card for German (as first language) and mathematics. The German grading system ranges from 1 (outstanding) to 6 (fail). To facilitate the interpretation of our results, school grades were reverse coded so that higher scores reflected more positive outcomes.

#### Reading Achievement and Decoding Speed

Reading achievement was measured with items from the German National Assessment of Educational Progress (Köller et al., 2010) and with items from the Study of Initial Achievement Levels and Academic Growth in Secondary Schools in the City of Hamburg in Grade 11 (Lehmann et al., 2004). The tasks consisted of four stimulus texts to which several questions (multiple-choice items, association tasks, open-ended, or half open-ended questions) had to be answered. Students had to extract information, check it for quality and relevance, and interpret it. They also had to detect intentions and derive conclusions from the text. We used sum scores for our analyses (α = 0.85).

Decoding speed was assessed with a test by Retelsdorf et al. (2012) that has been developed in accordance with the German PISA decoding speed test (Schneider et al., 2007). The students were asked to read a 740-word stimulus text and to identify numerals (e.g., “twenty,” “fifty-six”) by underlining them. In order to assess the students’ speed, the time limit was 2 min, which was not enough to complete the whole text. The number of read words was used as an indicator of decoding speed.

### Analyses

#### Factor Analyses

To test the dimensionality of the SRHI-R, we randomly split our sample into two data sets (*n*_1_ = 419, *n*_2_ = 999). We decided to randomly select a smaller subsample (ca. 30%) for an exploratory factor analysis (EFA) and a larger one (ca. 70%) for confirmatory factor analyses (CFA) due to the different sample size requirements of both analyses. EFA was performed with Maximum Likelihood Estimator and Promax rotation. We applied Promax rotation because if multiple dimensions would turn out at all, the assumption of uncorrelated factors did not seem plausible according to previous research on the structure of habit (Verplanken and Orbell, 2003; Verplanken et al., 2005). In the EFA the number of extracted factors has been determined using the scree test (Cattell, 1966). All factor analyses were estimated using Mplus, Version 6.11 using a robust full information likelihood estimator accounting for missing data (on average 3.4 % of the data were missing). Thus, we treated our data as continuously which should be feasible following Rhemtulla et al. (2012). They say that five point Likert-like scales in connection with a sufficient sample size allow treating item responses as continuous. Moreover, in research on predictors of reading achievement, this approach has traditionally been used, so the connectivity to related research seemed higher this way. Finally, inspection of the intraclass correlations (ICC) for all questionnaire items did not indicate the necessity of applying sandwich estimators (ICC ≤ 0.018).

To cross-validate the findings of this EFA, we applied CFA using the larger sample. Several indices of fit have been suggested to evaluate the goodness of fit for CFAs (e.g., Marsh, 2007; West et al., 2012). For the present analyses, we considered the root mean square error of approximation (RMSEA), the Tucker-Lewis Index (TLI), the Comparative Fit Index (CFI), and the standardized root mean square residual (SRMR). TLI and CFI values greater than 0.90 or 0.95 are typically interpreted to reflect an acceptable or excellent fit to the data. RMSEA values lower than 0.05, 0.06, or 0.08 and SRMR values lower than 0.08 or 0.10 are typically interpreted to reflect a close or a reasonable fit to the data.

#### Measurement Invariance

Measurement invariance for gender and thematic study profiles was tested by comparing nested models with different degrees of parameter restrictions (none, factor loadings, item intercepts, item residual variances). If the fit of the model with more restrictions (e.g., a model with intercepts, loadings, and residual variances held equal across groups) does not differ substantially from the model with less restrictions, a stronger form of invariance is be supported. We followed the *ad hoc* guidelines for an evaluation of model fit when testing for measurement invariance presented by Cheung and Rensvold (2002) and Chen (2007). Because the χ^2^-difference test tends to be unreliable in large samples these authors suggested that support for the more restrictive model requires a change in CFI of less than 0.01 or a change in RMSEA of less than 0.015.

## Results

### Dimensionality and Psychometric Properties of the SRHI-R

Using EFA, the scree test clearly indicated a unidimensional solution (first four Eigenvalues: 7.43, 0.49, 0.45, and 0.34) with the first factor explaining 79% of the variance. All items had substantial loadings (≥0.75) on the first factor (see **Table 1**). This result was cross-validated using CFA. The first model with all items loading on one factor did not fit the data sufficiently, χ^2^(35) = 494.92, CFI = 0.93, TLI = 0.90, RMSEA = 0.12, SRMR = 0.03. Due to high modification indices, we allowed correlations between residuals of two item pairs (three and five, four and six). These modifications seem to be theoretically sound as both item pairs measure a different aspect of automaticity. The modified model appeared to fit the data well: χ^2^(33) = 273.66; CFI = 0.96; TLI = 0.95; RMSEA = 0.08; SRMR = 0.02. All items loaded significantly (*p* < 0.001) and substantially (λ ≥ 0.70) on the latent factor (see **Table 1**). For all remaining analyses, we used the full sample.

Means, standard deviations, corrected item difficulties, and item selectivities for the unidimensional scale are provided in **Table 1**. The SRHI-R showed good to excellent corrected item difficulties (*M*_pcorr_ = 0.46, Range: 0.38 to 0.59)^1^ and good to excellent item selectivities (*r*_it_ ≥ 0.72). The internal consistency was excellent: Cronbach’s α = 0.96.

### Measurement Invariance

Models with different invariance constraints were compared to test measurement invariance. The least demanding model imposed no invariance constraints, the most demanding model posited invariance of factor loadings, item intercepts, and item residual variances. Following the *ad hoc* guidelines for an evaluation of model fit when testing for measurement invariance presented by Cheung and Rensvold (2002) and Chen (2007), the assumption of strict model invariance was supported for gender and partial strict model invariance was supported for thematic study-profiles (see **Table 2**). We also tested for group differences regarding gender and thematic study-profile applying a univariate ANOVA using the manifest means from the SRHI-R. We found a significant main effect for thematic study-profiles, *F*(4) = 4.74, *p* < 0.001, η^2^ = 0.01, and for gender, *F*(1) = 66.86, *p* < 0.001, η^2^ = 0.05; the interaction between both was not significant, *F*(4) = 1.56, *p* > 0.10. *Post hoc* analyses for thematic study-profiles showed that students in the language and esthetics profiles reached higher scores than students in the science, social science, and sports profiles.

**Table 2:** Measurement invariance across gender and thematic study-profiles.

Model	Parameters constrained	χ^2^	*df*	CFI	|ΔCFI|	TLI	RMSEA	|ΔRMSEA|	SRMR
Measurement invariance across gender									
1	None (configural invariance)	402.74	66	0.959	-	0.944	0.085	-	0.025
2	FL (metric invariance)	447.40	75	0.955	0.004	0.946	0.084	0.001	0.038
3	FL, II (scalar invariance)	526.55	84	0.947	0.008	0.943	0.087	0.003	0.042
4	FL, II, IRV	532.98	94	0.947	0.000	0.949	0.082	0.005	0.049
Measurement invariance across thematic study-profiles									
1	None (configural invariance)	487.94	165	0.962	-	0.949	0.084	-	0.028
2	FL (metric invariance)	549.86	201	0.959	0.003	0.955	0.079	0.005	0.042
3	FL, II (scalar invariance)	655.36	237	0.951	0.008	0.954	0.080	0.001	0.049
3a	FL, partial II	595.51	225	0.957	0.002	0.957	0.077	0.002	0.045
4	FL, partial II, IRV	634.60	265	0.957	0.000	0.964	0.071	0.006	0.051
4a	FL, partial II, partial IRV	624.37	261	0.958	0.001	0.964	0.071	0.000	0.049

*FL, factor loadings, II, item intercepts, IRV, item residual variances. For model identification in model 1 and 2 (item intercepts freely estimated) latent means were fixed to zero. Changes in CFI < 0.01 and RMSEA < 0.015 indicate a reasonable change in model fit. ΔCFI and ΔRMSEA represent the difference in model fit compared to the less restricted model.*

### Correlations with External Criteria

Regarding the correlations between the SRHI-R and the external criteria our assumptions were by and large corroborated. In detail, we found strong correlations between the SRHI-R and reading frequency (*r* = 0.69, *p* < 0.001). In order to avoid an overestimation of correlations, for the estimation of these correlations, we did not take items measuring history of repetition (Item 1 and Item 7) into account, because these items directly refer to reading behavior. Moreover intrinsic reading motivation correlated highly (*r* = 0.85, *p* < 0.001) and reading self-concept moderately (*r* = 0.32, *p* < 0.001) positive with the SRHI-R. To investigate whether intrinsic reading motivation and reading habit measure two separate constructs, we compared a model with all items from both measures namely the SRHI-R and the intrinsic reading motivation subscale loading on one single factor [χ^2^(88) = 2086.41; CFI = 0.87; TLI = 0.84; RMSEA = 0.13; SRMR = 0.05] to a two-dimensional model representing the two constructs separately [χ^2^(87) = 848.50; CFI = 0.95; TLI = 0.94; RMSEA = 0.07; SRMR = 0.04]. The data show a significantly better fit to the two-dimensional model (Δχ^2^ = 1237; *df* = 1; *p* < 0.001) indicating that the items for the SRHI-R and intrinsic reading motivation measure different constructs.

The correlation between the SRHI-R and the verbal self-concept (*r* = 0.26, *p* < 0.001) was substantially larger than for mathematics self-concept (*r* = –0.02, *ns*). This held true for the school grades in German (*r* = 0.21, *p* < 0.001) and mathematics (*r* = 0.02, *ns*) as well. Finally, reading achievement (*r* = 0.22, *p* < 0.001) and decoding speed (*r* = 0.18, *p* < 0.001) correlated moderately with the SRHI-R.

### SRHI-R and Reading Frequency Predicting Reading Achievement and Decoding Speed

We used a series of regression models to test if the SRHI-R explains variance in reading achievement and decoding speed over and above reading frequency. The results presented in **Table 3** clearly indicated that the SRHI-R led to higher amounts of explained variance in both reading skills (see Model 2 for each achievement measure). Moreover, reading frequency did not significantly predict reading achievement and decoding speed when the SRHI-R was also used as a predictor. Due to the high correlation between the SRHI-R and intrinsic reading motivation, we tested an additional model also including intrinsic reading motivation as a predictor of reading achievement and decoding speed. Intrinsic reading motivation turned out as a significant predictor of both achievement measures again increasing the amounts of explained variance. Thereby, the SRHI-R remained a significant predictor only for decoding speed but not for reading achievement.^2^

**Table 3:** Regression models predicting reading achievement and decoding speed by reading frequency, the SRHI-R and intrinsic reading motivation.

	Reading achievement						Decoding speed					
	Model 1		Model 2		Model 3		Model 1		Model 2		Model 3	
	β	*P*	β	*p*	β	*p*	β	*p*	β	*p*	β	*p*
Reading frequency	0.16	0.000	0.02	0.577	-0.02	0.622	0.11	0.000	-0.02	0.624	-0.04	0.314
SRHI-R^a^	-	-	0.21	0.000	0.07	0.14	-	-	0.19	0.000	0.12	0.017
Intrinsic reading motivation	-	-	-	-	0.21	0.000	-	-	-	-	0.11	0.019
	
*R*^2^_adjusted_	0.026		0.048		0.062		0.012		0.029		0.032	
Change in *R*^2^			M1 vs. M2: *p* < 0.001		M2 vs. M3: *p* < 0.000				M1 vs. M2: *p* < 0.001		M2 vs. M3: *p* < 0.05	

*^a^Items dealing with reading frequency were omitted from the SRHI-R to prevent overestimation. Using the full SRHI-R led to almost identical results.*

## Discussion

Reading habit plays an important role in reading research (e.g., Wang and Guthrie, 2004; Schiefele et al., 2012). Although many studies deal with this topic or related research questions, the theoretical fundamentals of reading habit have seldom been discussed. Researchers mostly use simple measures of behavioral frequency to capture habit. Following discussions (Verplanken and Orbell, 2003) on how to understand and measure habit in general (i.e., not particularly in the reading domain), we aimed to introduce the idea that habit is more than just behavioral frequency to reading research. Therefore, we adapted the well-established SRHI (Verplanken and Orbell, 2003) to reading research.

As was well supported for the original measure (Verplanken et al., 2005) the SRHI-R turned out to be unidimensional. Even though, small modifications were necessary to obtain a good model fit for the unidimensional CFA, the empirical evidence for this model seems reasonable. First, the results of the EFA clearly indicated a single factor solution with nearly 80% of the variance explained by the first factor. Second, when evaluating the model fit of the non-modified unidimensional CFA, the RMSEA is the only index suggesting a bad model fit, while CFI, TLI, and SRMR meet the cut-off criteria given as thumb rules in the literature. Third, theoretically habit is understood as a unidimensional psychological construct comprising broad content. Thus, we feel that the small hints on possible multidimensionality of reading habit in our study should not be overrated. At least, we are convinced that it is feasible to assume essential unidimensionality for the SRHI-R (see e.g., Slocum-Gori et al., 2009 for a discussion of essential dimensionality).

The SRHI-R showed good psychometric properties. Moreover, the invariance of the measurement model was supported for two sets of subpopulations (gender and thematic study-profiles), indicating that it was valid for these groups. In order to obtain indicators of external validity, we investigated correlations with a set of external criteria. As expected, reading frequency and the SRHI-R correlated strongly. Due to the high correlation, we investigated the predictive power of the SRHI-R compared to reading frequency. As expected, reading habit was correlated positively with achievement outcomes, namely reading achievement and decoding speed. For both, the SRHI-R turned out to be a better predictor. When also including intrinsic reading motivation as a predictor the findings become somewhat more differentiated. Predicting reading achievement, the effect of the SRHI-R vanishes indicating that the habit effect may be explained by higher motivation. However, predicting decoding speed, the effect of SRHI-R remained significant when including intrinsic reading motivation. These differential relations of habit and motivation with different aspects of reading cannot be explained in detail with the current study. However, it seems plausible that for different aspects of reading different determinants are important, since we know from previous research that different reading skills vary in their development (e.g., Bast and Reitsma, 1998; Aunola et al., 2002; Retelsdorf et al., 2012). Thereby, habits may be more important for rather basic reading skills such as decoding speed than for more complex reading skills as measured by our reading achievement test. For such basic skills high amounts of reading practice may be particularly relevant since they benefit from high levels of automatization (cf. Stanovich, 1986). Such high amounts of practice as well as automatization are essentials of reading habit as, we understand it. Despite the different results for reading achievement and decoding speed, these findings highlight the fact that the scale could make a contribution to the research on prerequisites of reading outcomes. Moreover, given the differential unique contributions of motivation and habit to decoding speed, we think that both variables indeed measure unique constructs.

Finally, the differential correlations with school grades in German as the first language and mathematics as well as verbal and mathematics self-concepts corroborated our assumptions. All in all, the correlations and group comparisons yielded the expected results supporting the validity of the SRHI-R. As an economic measure, we hold the SRHI-R to be a useful measure even for longitudinal large-scale assessments.

Next to these hints toward sufficient quality criteria of the SRHI-R, we again want to stress two advantages compared to typical measures of reading habit. First, the reliability can be expected to be higher in comparison to the typical one- or two-item measures. Second, and most importantly, the SRHI-R is embedded in a theoretical framework of habit so that it may reflect a more elaborated understanding of habit. This deeper understanding of reading habit may also stimulate new research regarding antecedents and consequences of reading habit.

### Limitations and Future Directions

Finally, some limitations of the present study need to be addressed. First, our sample only comprised students from the upper secondary academic track. Large means and maybe somewhat restricted variance in the study variables may have affected the correlational results of our study. Second, in accordance with the work of Allen et al. (1992) and Keatley et al. (2015), research on the differences between the various measures assumed to capture reading habit is needed. We assume that integrating the various methods may lead to an even more comprehensive picture of the habit concept. Finally, one may argue that the quite high correlation between reading habit and frequency serves as a good argument for moving forward with measuring just frequency even though using regression analyses the SRHI-R showed to be a better predictor of decoding speed and reading achievement. Still, it is important to note, that we do not assume the SRHI-R to replace measures of behavioral frequency but to be utilized for the purpose of measuring reading habit which requires a measure that takes the theoretical foundation of the construct into account.

Despite these limitations, we hope to have offered some new impulses for research on reading habit with our research. The questionnaire seems to be a valid and reliable measure of reading habit covering more than just behavioral frequency.

## Author Contributions

Both authors did contribute substantially to the conception and design of the work. JR was predominantly responsible for the acquisition of the data. FS did the majority of the analyses and interpretation of the data. FS drafted the work and JR revised it critically for important intellectual content. Both authors give final approval of the version to be published and agree to be accountable for all aspects of the work and ensure that questions related to the accuracy or integrity of any part of the work are appropriately investigated and resolved.

## Conflict of Interest Statement

The authors declare that the research was conducted in the absence of any commercial or financial relationships that could be construed as a potential conflict of interest.

## Footnotes

1 The item difficulties have been calculated following Fisseni (1997). For this index the squared most extreme answer is divided by the mean of the squared given answers, thus taking the variance of the answers into account.

2 Tests for multicollinearity indicated that the variance inflation factor (VIF ≤ 2.98) and the tolerance (≥0.34) were far from indicating problematic amounts of multicollinearity. A VIF of larger than 5.00 or 10.00 or a tolerance of less than 0.20 or 0.10 suggest multicollinearity problems, even though these rules of thumb should only be used as rough guidelines (cf. Cohen et al., 2003).
